# Supplementary material for: Exploring Social and Financial Hardship, Mental Health Problems and the Role of Social Support in Asylum Seekers Using Structural Equation Modelling
Source: Int J Environ Res Public Health. 2020 Sep 23;17(19):6948. doi: 10.3390/ijerph17196948 (PMC7579644; doi:10.3390/ijerph17196948)
Supplement: Supplementary file 1 [file ijerph-17-06948-s001.pptx]

## Slide 1
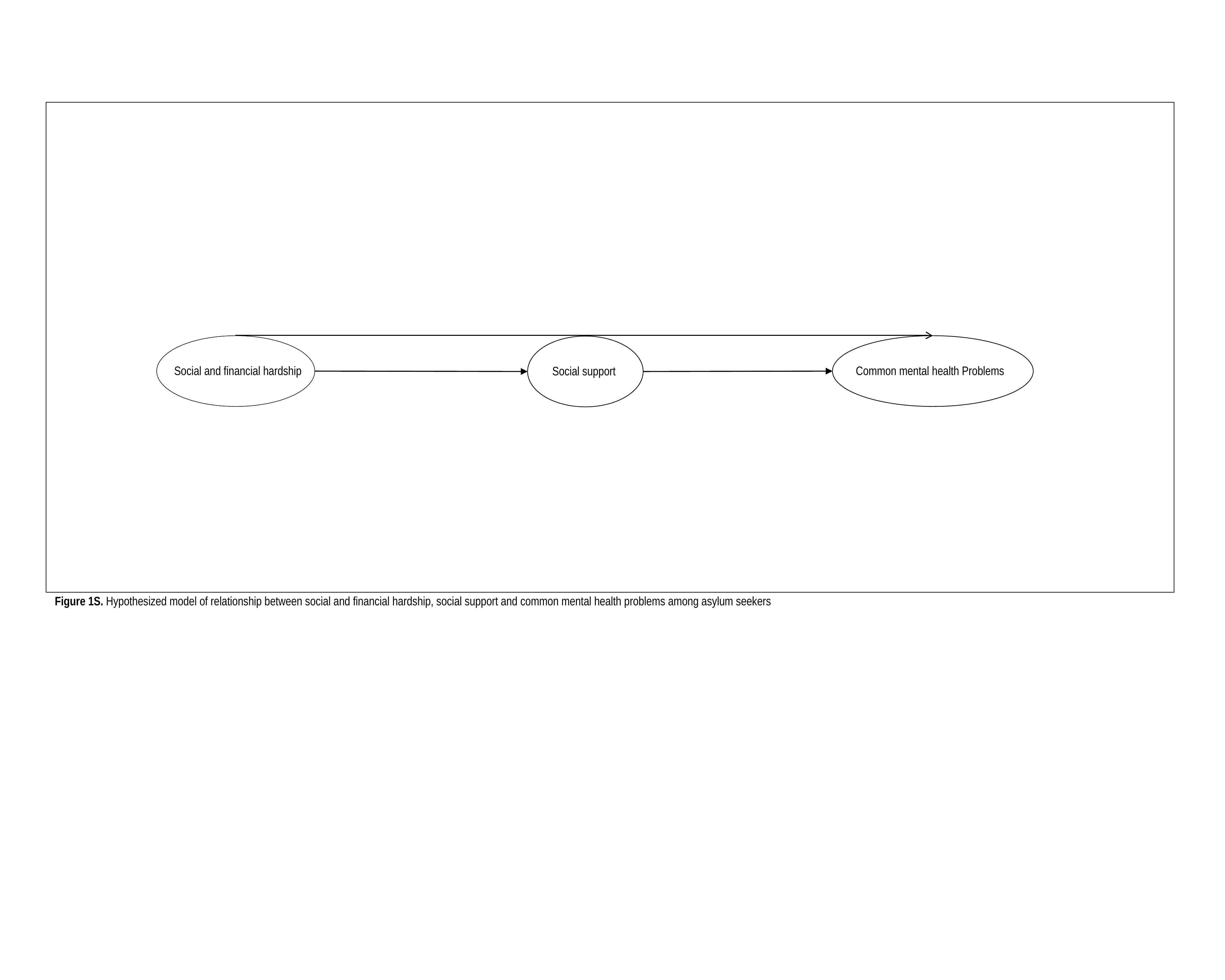

Social and financial hardship
Common mental health Problems
Social support
Figure 1S. Hypothesized model of relationship between social and financial hardship, social support and common mental health problems among asylum seekers
